# Prevalence of ADHD in nonpsychotic adult psychiatric care (ADPSYC): A multinational cross-sectional study in Europe

**DOI:** 10.1186/s12888-015-0624-5

**Published:** 2015-10-13

**Authors:** Walter Deberdt, Johannes Thome, Jeremie Lebrec, Susanne Kraemer, Irene Fregenal, J. Antoni Ramos-Quiroga, Muhammad Arif

**Affiliations:** NV Eli Lilly Benelux, Markiesstraat 1, 1000 Brussels, Belgium; Department of Psychiatry, University of Rostock, Gehlsheimer Str. 20, D-18147 Rostock, Germany; Lilly Deutschland GmbH, Werner Reimers-Str. 2-4, 61352 Bad Homburg, Germany; Medical Liaison, Lilly Spain, Avenida de la Industria, 30. Alcobendas, E-28108 Madrid, Spain; ADHD Program, Department of Psychiatry, CIBERSAM Hospital Universitari Vall d’Hebron Universitat Autònoma de Barcelona, Pg. Vall d’Hebron, 119-129 Barcelona, Spain; Leicester (Adult) ADHD Service, Leicestershire Partnership NHS Trust, Leicester, United Kingdom

**Keywords:** Attention-deficit/hyperactivity disorder, Adult, Psychiatric outpatients, Prevalence, Europe

## Abstract

**Background:**

Attention-deficit/hyperactivity disorder (ADHD) often persists into adulthood.

This study was designed to estimate the prevalence of ADHD in adult psychiatric outpatients in several European countries.

**Method:**

ADHD diagnosis was made using the Diagnostic Interview for ADHD in Adults, version 2.0 (DIVA), according to criteria of the Diagnostic and Statistical Manual of Mental Disorders, 4th Edition, Text Revision (DSM-IV-TR) and 5th Edition (DSM-5).

**Results:**

Of 5662 patients present/approached, 2284 (40.3 %) consented, of whom 1986 patients (87.0 %) completed the study. Based on the DIVA, and applying DSM-IV-TR or DSM-5 criteria, 15.8 % (95 % confidence interval [CI] 14.2 %-17.4 %) or 17.4 % (95 % CI 15.7 %-19.0 %) of patients were diagnosed with ADHD, respectively. The prevalence of ADHD was 15.3 % when counting as non-ADHD those patients who screened positive but did not complete the DIVA (DSM-5).

**Conclusions:**

Estimates from this study indicate that a relevant part of the psychiatric outpatient care population suffers from ADHD.

## Background

There is growing recognition that undiagnosed and untreated attention-deficit/hyperactivity disorder (ADHD) can result in multiple negative consequences for the individual’s life as well as for society. Adults with ADHD tend to suffer from major socioeconomic disadvantage [1], functional impairment [2], and a diminished quality of life [3–5]. Lower levels of education, higher levels of unemployment, and substance use, are known to be significantly associated with ADHD in adults [6]. Accidents [7], early parenthood [8], and difficulties in governing financial issues [9] are thought to be more prevalent in adults with ADHD than in the general population. ADHD is much more prevalent in the prison population compared with nondelinquent controls [10, 11]. Among patients with ADHD, the rate of criminality is lower when they are receiving ADHD medication [12].

ADHD is also associated with a number of comorbid psychiatric disorders, such as mood, anxiety, and substance use disorders [13]. Knowing the prevalence of ADHD in adult outpatient psychiatric care could help clinicians take this treatable disorder into consideration when evaluating their patients, enabling them to target both ADHD and comorbid condition (s), and also to consider ADHD as a possible differential diagnosis.

There are a number of studies available regarding the prevalence of ADHD in the general population. For example, a meta-analysis by Simon and colleagues [14] determined the pooled prevalence of adults with ADHD to be 2.5 % (95 % CI, 2.1 %-3.1 %). Slightly higher estimates of 3.4 % [13] and 4.4 % [15] have also been proposed.

Prevalence of ADHD is expected to be higher in the psychiatric outpatient population than in the general population, but only a few estimates are available. Almeida Montes and colleagues [16] found in a psychiatric nonpsychotic outpatient sample in Mexico a prevalence of 16.8 %. Similarly, Rao and Place [17] estimated the frequency of undiagnosed ADHD in 4 general adult psychiatry outpatient clinics in North East England to be 22 %. Therefore, to contribute to a better understanding of the pattern of clinical presentation of ADHD in adult mental health care, the primary objective of this study was to estimate the prevalence of ADHD in adult outpatient psychiatric care in several European countries. Secondary objectives of this study were to 1) characterize the adult ADHD population in terms of symptoms, functioning, quality of life, work status, resource use and comorbid conditions, and 2) to examine the effect of differences between Diagnostic and Statistical Manual of Mental Disorders, 4th Edition, Text Revision (DSM-IV-TR) [18] and 5th Edition (DSM-5) [19] criteria for ADHD.

## Methods

### Site selection and patients

This was a multinational study conducted in Austria, Belgium, Denmark, Germany, The Netherlands, Spain, Sweden, and the United Kingdom. The study aim was to reflect the situation and presence of adult patients with ADHD in the psychiatric outpatient health care system in each country or a pool/group of countries with a similar mental health care organization. Therefore, the study was not conducted at specialized ADHD centres, but at sites providing general psychiatric care to a population as unselected as possible in the respective health care system. Four types of sites were defined to achieve this goal: general psychiatry outpatient clinics linked to general hospitals, private psychiatric practices, community mental health centres, and outpatient clinics of psychiatric hospitals. The distribution of sites was intended to reflect the different levels and various settings of outpatient mental health care in each participating country and to examine a representative population of adults using outpatient psychiatric care. However, for some countries, because of the limited number of sites able to participate, the results might not be as representative as intended.

At each study site, all patients, regardless of their existing diagnoses (including ADHD), were invited to participate in the study. The sites were asked to approach all patients during their normal clinic days during prespecified times and days of the week until their predefined number of entered patients per week was reached. These prespecified inclusion periods varied over the course of the study to cover all working days and times (to ensure that a representative population of that specific facility was included). Inclusion criteria were 1) males or females attending psychiatric outpatient care, ages 18 to 65 years; and 2) having signed an informed consent to release information prior to any procedure. Exclusion criteria were 1) mental disability or disease state to an extent that prevents the patient from understanding the nature of the study or that prevents the patient from reliably following procedures; and 2) psychotic disorder at presentation or from patient’s history (schizophrenia, schizo-affective, schizophreniform, delusional disorder), however, treatment with antipsychotics for other indications was not an exclusion criterion.

This study was submitted to ethical review boards (ERBs) for approval whenever required by local law. In addition, regardless of local law, this study was submitted to at least one independent body (for example, ERB) per country for review and to confirm that the study was considered noninterventional in that country. Regulatory authorities were notified and approval sought as required by local laws and regulations. Progress reports were submitted to ERBs and regulatory authorities as required by local laws and regulations. This study was conducted in accordance with the ethical principles that have their origin in the Declaration of Helsinki and that are consistent with good clinical practice (GCP) and applicable laws and regulations of the countries where the study was conducted.

### Study design, procedures, and scales

After having provided their informed consent for the release of their anonymized data, patients were assessed clinically, using solicited questions and diagnostic instruments (self–and physician-rated scales). Screening procedures closely followed the guidance given in the European Consensus Statement of the European Network Adult ADHD [20]. After collection of patient demographic information and characteristics, patients were asked about any previous ADHD diagnosis. Patients were also screened with Part A of the Adult ADHD Self-Report Scale (ASRS) [21]. (The ASRS is an instrument consisting of the 18 DSM-IV-TR criteria. Six of the 18 questions, found to be the most predictive of ADHD, form Part A.) As the wording of 4 of the items of the ASRS Part A is identical to the 4 ADHD items in the Provisional Diagnostic Instrument-4 (PDI-4) [22], responses on these items were used to derive a score for the PDI-4 as well.

A positive screen according to the ASRS (at least 4 out of 6 responses exceeding threshold) or the PDI-4 ADHD questions (at least 3 out of 4 responses exceeding threshold), a previous ADHD diagnosis, or suspicion of ADHD by clinical impression resulted in further assessment using the Diagnostic Interview for ADHD in Adults (DIVA) [23] according to the criteria of the DSM-IV-TR and DSM-5. For DSM-5, the number of symptoms required for a diagnosis of ADHD (from either the inattention criteria or the hyperactivity/impulsivity criteria, or both) has been lowered from 6 to 5. Also, ADHD symptoms must have been present by age 12 years, compared to 7 years for DSM-IV-TR. The DIVA is a DSM-based semistructured interview covering the childhood and adulthood DSM symptoms list for ADHD, and providing examples of impairments commonly associated with the symptoms in 5 areas of everyday life for each age group: work and education; relationships and family life; social contacts; free time and hobbies; self-confidence and self-image. Whenever possible, the DIVA was completed with the patient in the presence of a partner/friend and/or family member, to enable retrospective and collateral information to be ascertained at the same time. Information received via telephone from partners/friends and/or family members was also accepted, if available. Diagnosis of psychiatric disorders other than ADHD was done according to the usual standards of each site.

The following scales/questions were given to all subjects, regardless of the outcome of ADHD screening. Clinical Global Impressions-Severity (CGI-S) [24], a clinician-rated scale ranging from 1 (not at all ill) to 7 (among the most extremely ill), was used to rate the severity of overall mental illness at the time of study entry. Information about clinical status (other than ADHD) and medical history (guided by DSM-IV-TR or International Statistical Classification of Diseases and Related Health Problems, 10th Revision, whichever is used routinely at that site) was collected along with family history. Information on functioning was collected using the Sheehan Disability Scale (SDS) [25] as well as other questions to the patient. The SDS was used to assess changes in patients’ personal work schedules, social life/leisure activities, and family life/home responsibilities. Responses for each item range from 0 to 10 (higher values indicate greater disruption). The SDS also asks for the number of days lost and unproductive days in the last week. Information on the patients’ quality of life (QoL) and resource utilization was collected by the use of EuroQol-5 Dimensions (EQ-5D) [26] as well as direct questions to the patient. The EQ-5D assesses a patient’s current, perceived, health-related quality of life by asking the patient to rate their impairment as low, medium, or high. The EuroQol Visual Analog Scale (EQ VAS) assesses the respondent’s self-rated overall health status on the day of completion (0 = lowest possible to 100 = highest possible).

In addition, patients were asked questions covering known ADHD related aspects concerning general health and conduct of life.

### Statistical analysis

Since the analyses were largely descriptive and exploratory, p-values were not calculated. Confidence intervals (95 % level, 2-sided, computed using the normal approximation) were used as descriptive measures only and were not meant to support statistical inferences. No correction for multiplicity was done.

The prevalence of ADHD in outpatient psychiatric care was estimated using the DIVA. The denominator comprised all patients who completed the screening instrument, excluding any who had a positive screen but were not assessed using the DIVA. A sensitivity analysis was also completed to assess the effect of excluding these patients.

The association of ADHD diagnosis with a subset of endpoints was analyzed using regression models (either logistic regression or analysis of covariance, as appropriate), adjusting for age, gender, and country.

## Results

### Patient disposition and characteristics

**Fig. 1:**
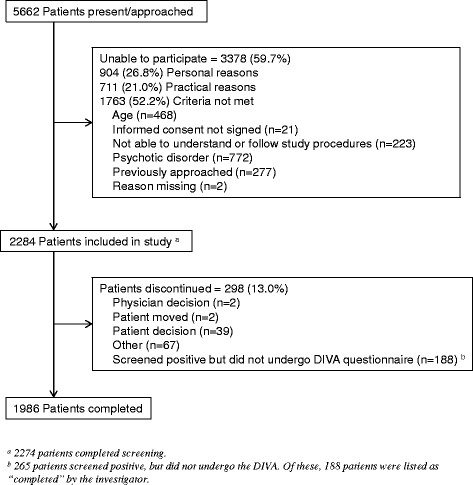
Patient disposition. Abbreviations: DIVA = Diagnostic Interview for ADHD in Adults; n = number of affected patients

Of 5662 outpatients present at sites during the study and approached, 2284 (40.3 %) were included. Reasons for ineligibility in the study are listed in Fig. 1. Patients were 17 to 72 (median = 42) years of age, and 58.8 % were women. Of the 2284 patients included in the study, 1079 (47.2 %) screened positive for ADHD. Table 1 provides the number of the patients fulfilling the various ADHD screening criteria and the percentages of patients eventually diagnosed with ADHD according to the DIVA.

**Table 1:** Screening Criteria Versus DIVA Completion

Criteria	Positive Screen	Screened Positive But Did Not Complete the DIVA	ADHD Established by the DIVA
	(*N* = 1079)	(*N* = 265)	(*N* = 349)
ASRS	941 (87.2)	244 (25.9)^a^	313 (89.7)
PDI-4	433 (40.1)	74 (17.1)^a^	195 (55.9)

Abbreviations: *ADHD* = attention-deficit/hyperactivity disorder; *ASRS* = Adult ADHD Self-Report Scale, DIVA; *DIVA* = Diagnostic Interview for ADHD in Adults; *N* = number of patients; *PDI*-4 = Provisional Diagnostic Instrument-4

^a^Percentage of patients who screened positive but did not receive the DIVA

### Prevalence of ADHD in adults

Based on the DIVA (using DSM-IV-TR criteria), 15.8 % (318/2009, 95 % confidence interval [CI] 14.2 %-17.4 %) of all participating patients suffered from ADHD (Table 2). The majority of these patients exhibited the combined type (63.2 %); 29.6 % were of the inattentive type, and 7.2 % were of the hyperactive-impulsive type. Reducing the required number of symptoms in childhood from 6 to 5 resulted in an increase in prevalence to 19.9 %; this is in fact close to the clinical judgment (last DIVA item: “Diagnosis ADHD yes/no”) of 22.6 %. When DSM-5 criteria were applied to patient responses on the DIVA, the prevalence of ADHD was 17.4 % (349/2009; 95 % CI, 15.7 %-19.0 %). The prevalence of ADHD was 15.3 % (349/2274; 95 % CI, 13.9 %-16.8 %) when counting as non-ADHD those patients who screened positive but did not complete the DIVA (sensitivity analysis). All statistics presented going forward will be based on DSM-5 criteria.

**Table 2:** Prevalence of ADHD According to the DIVA Using DSM-IV-TR and DSM-5 Criteria

	Eligible Patients (*N* = 2284)	
	*n* (%)	
Patients completing screening	2274/2284 (99.6)^a^	
Positive screen for ADHD	1079/2274 (47.4)	
Negative screen for ADHD	1195/2274 (52.6)	
Patients completing DIVA	814/1079 (75.4)^b^	
	DSM-IV-TR Criteria	DSM-5 Criteria
	*n* (%)	*n* (%)
	*N* = 2284^c^	*N* = 2284^c^
Diagnosed with ADHD based on DIVA	318 (15.8)	349 (17.4)
95 % CI	14.2-17.4	15.7-19.0
Diagnosis by category		
Combined	201 (10.0)	256 (12.7)
Inattentive	94 (4.7)	78 (3.9)
Hyperactive/impulsive	23 (1.1)	15 (0.7)

Abbreviations: *ADHD* = attention-deficit/hyperactivity disorder; *CI* = confidence interval; *DIVA* = Diagnostic Interview for ADHD in Adults; *DSM* = *Diagnostic and Statistical Manual of Mental Disorders*; IV = 4th Edition; *N* = number of patients; *n* = number of affected patients

^a^10 patients who entered the study discontinued before completing the screening procedure

^b^265 patients who screened positive were not given the DIVA

^c^The calculation of prevalence was based on 2009 patients (2284 patients minus 10 patients who did not complete the screening, and 265 patients who screened positive but were not given the DIVA)

Outpatients with ADHD were younger compared to patients without ADHD for both males (median 32 versus 45 years old) and females (median 34 versus 43 years old). Of the 349 patients who were diagnosed with ADHD using the DIVA, 53.9 % had not been previously diagnosed with ADHD. Most of the patients who were diagnosed with ADHD with the DIVA had not been treated for ADHD in childhood (93.1 %), adolescence (90.8 %), or adulthood (67.3 %). Among patients with a known ADHD diagnosis prior to the study, average age at diagnosis was 28.1 (standard deviation 13.62) years.

**Fig. 2:**
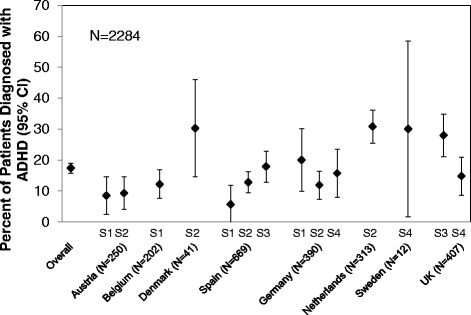
ADHD prevalence in nonpsychotic psychiatric outpatients as determined by the DIVA according to criteria of the DSM-5, by country and setting. Abbreviations: ADHD = attention-deficit/hyperactivity disorder; CI = confidence interval; DIVA = Diagnostic Interview for ADHD in Adults; DSM-5 = Diagnostic and Statistical Manual of Mental Disorders, 5th Edition; N = number of patients; S1 = general psychiatry outpatient clinics linked to general hospitals; S2 = private psychiatric practices; S3 = community mental health centers; S4 = outpatient clinics of psychiatric hospitals; UK = United Kingdom

Considerable variability in ADHD rates was associated with country (range: 9.0 % to 30.8 %) (Fig. 2). This variability was also evident when countries were pooled by region: Austria/Belgium/Germany (12.3 % [95 % CI, 9.9 %-14.6 %]); Spain (14.1 % [95 % CI, 11.4 %-16.7 %]); United Kingdom (22.4 % [95 % CI, 17.5 %-27.2 %]); and Denmark/Sweden/The Netherlands (30.7 % [95 % CI, 25.8 %-35.7 %]). Differences in ADHD rates were also found between outpatient settings: general psychiatry outpatient clinics linked to general hospitals (11.7 % [95 % CI, 8.5 %-14.9 %]), private psychiatric practices (18.0 % [95 % CI, 15.7 %-20.4 %]), community mental health centres (22.1 % [95 % CI, 18.0 %-26.2 %]), and outpatient clinics of psychiatric hospitals (15.8 % [95 % CI, 10.9 %-20.7 %]). Rates of exclusion also varied considerably by country. The lowest exclusion rates were in Swedish (0 %; ADHD prevalence 30.0 %) and in Belgium (17.2 %; ADHD prevalence 12.2 %); the highest exclusion rates were in Austria (77.4 %; ADHD prevalence 9 %) and Germany (71.3 %; ADHD prevalence 14.2 %).

The prevalence of ADHD in women was less than in men (14.4 % [95 % CI, 12.4 %-16.4 %] versus 21.6 % [95 % CI, 18.8 %-24.4 %]). However, a greater percentage of women than men were diagnosed with ADHD for the first time during the study (60.4 % versus 47.8 %). Patients with ADHD were more likely to have a first-degree relative diagnosed with ADHD than patients without ADHD (14.9 % versus 3.7 %).

### Psychiatric diagnoses and neurological symptoms

The most common type of comorbid psychiatric disorder among patients with ADHD was depression. Within the ADHD group, depression was more commonly diagnosed among patients who were diagnosed with ADHD during the study (48.9 % [95 % CI, 41.8 %-56.1 %]) than among patients whose ADHD was known prior to the study (ADHD treated: 36.0 % [95 % CI, 27.2 %-44.8 %]; ADHD untreated: 36.2 % [95 % CI, 22.4 %-49.9 %]). Anxiety disorder was also common among ADHD and non-ADHD patients (Table 3). Anxiety disorders tended to be less prevalent among patients who had ADHD that was previously identified and treated (28.1 % [95 % CI, 19.8 %-36.3 %]) compared to those patients with previously identified but untreated ADHD (44.7 % [95 % CI, 30.5 %-58.9 %]). Patients with ADHD, as compared to patients without ADHD, were more likely to present with substance use/dependence (Table 3). Interestingly, substance use was less common among patients with newly diagnosed ADHD (4.8 % [95 % CI, 1.7 %-7.8 %]) compared with patients previously treated for ADHD (14.9 % [95 % CI, 8.4 %-21.5 %]).

**Table 3:** Prevalence of Neurological Symptoms/Diagnoses and Psychiatric Disorders Among Patients with ADHD

	ADHD Diagnosis Established by the DIVA		
	Prevalence of ADHD Among Patients with Other Disorders	Prevalence of Other Disorders Among Patients with ADHD	Prevalence of Other Disorders Among Non-ADHD Patients
	(*N* = 349),	(*N* = 349),	(*N* = 1660),
	*n* (%)	*n* (%)	*n* (%)
Psychiatric disorders^a^			
Depression (*n* = 1215)	150 (12.3)	150 (43.0)	894 (53.9)
Dysthymia (*n* = 253)	34 (13.4)	34 (9.7)	198 (11.9)
Bipolar disorder (*n* = 268)	29 (10.8)	29 (8.3)	192 (11.6)
Obsessive-compulsive disorder (*n* = 126)	17 (13.5)	17 (4.9)	92 (5.5)
Anxiety disorders (*n* = 803)	127 (15.8)	127 (36.4)	575 (34.6)
Eating disorders (*n* = 113)	24 (21.2)	24 (6.9)	76 (4.6)
Substance abuse (*n* = 104)	32 (30.8)	32 (9.2)	56 (3.4)
Substance dependence (*n* = 86)	21 (24.4)	21 (6.0)	50 (3.0)
Alcohol dependence (*n* = 109)	14 (12.8)	14 (4.0)	81 (4.9)
Alcohol abuse (*n* = 133)	36 (27.1)	36 (10.3)	87 (5.2)
Non-substance dependence (*n* = 30)	4 (13.3)	4 (1.1)	20 (1.2)
Antisocial personality disorders (*n* = 24)	4 (16.7)	4 (1.1)	14 (0.8)
Borderline personality disorders (*n* = 172)	32 (18.6)	32 (9.2)	111 (6.7)
Autistic spectrum disorder (*n* = 36)	9 (25.0)	9 (2.6)	22 (1.3)
Other (*n* = 273)	56 (20.5)	56 (16.0)	184 (11.1)
No other psychiatric diagnosis (*n* = 115)	40 (34.8)	40 (11.5)	56 (3.4)
Neurological symptoms/diagnoses			
Any present		18 (5.2)	60 (3.6)
Tic disorder		7 (2.0)	29 (1.7)
Tourette’s syndrome		3 (0.9)	15 (0.9)
Coordination deficiencies		9 (2.6)	25 (1.5)
Deficiencies in fine motor skills		8 (2.3)	41 (2.5)

Abbreviations: *ADHD* = attention-deficit/hyperactivity disorder; *CI* = confidence interval; *DIVA* = Diagnostic Interview for ADHD in Adults; *N* = number of patients; *n* = number of affected patients

^a^The n’s for overall comorbidity rates in column 1 are based on all eligible patients (including those with a positive screen but no DIVA assessment) and do not add up to n’s in columns 3 and 4

Table 3 also summarises the percentages of patients with various disorders who also had ADHD. A diagnosis of ADHD was very common among patients presenting with substance abuse (30.8 % [95 % CI, 21.9 %-39.6 %]), substance dependence (24.4 % [95 % CI, 15.3 %-33.5 %]), and alcohol abuse (27.1 % [95 % CI, 19.5 %-34.6 %]).

Only 11.5 % [95 % CI, 8.1 %-14.8 %] of ADHD patients in the psychiatric outpatient settings were not diagnosed with any additional psychiatric diagnosis, an indication of the generally high disease burden in this condition. For those patients previously treated for ADHD, 18.4 % [95 % CI, 11.3 %-25.5 %] had no other psychiatric diagnosis; for patients with previously-identified but untreated ADHD, 23.4 % [95 % CI, 11.3 %-35.5 %] had no other psychiatric diagnosis.

Overall, neurological symptoms/diagnoses were rare and were not more common in patients with ADHD than without ADHD with only minor differences between groups in the percentage of patients with tics, Tourette’s syndrome, coordination deficiencies, or deficiencies in fine motor skills (Table 3).

Patients with ADHD reported more tobacco use than patients without ADHD (47.3 % [95 % CI, 42.0 %-52.5 %] versus 36.9 % [95 % CI 34.5 %-39.2 %]) as well as more respiratory dysfunction (12.3 % versus 6.3 %). Diagnoses related to higher age were less common in patients with ADHD (diabetes mellitus: 1.4 % versus 4.3 %; cardiovascular disease: 4.0 % versus 9.0 %).

### Resource utilization (treatment)

Treatment with psychotropic non-ADHD medications is substantial for both groups. Logistic regression analysis indicated that patients with ADHD were less likely to be prescribed antidepressants than patients who did not have ADHD (Table 4). However, patients with treated ADHD were more likely to be prescribed antipsychotic medication (17 out of 114 treated patients versus 1 out of 47 untreated patients).

**Table 4:** Resource Utilization: Psychiatric Medication/Therapy in the Previous 6 Months

	ADHD Diagnosis Established by the DIVA	ADHD Diagnosis Not Established	Odds Ratio (ADHD versus No ADHD)
	(*N* = 349)	(*N* = 1660)	(95 % CI)
Antidepressants, *n* (%)	199 (57.0)	1193 (71.9)	0.66 (0.49-0.89)
Anxiolytics, sedatives, and hypnotics, *n* (%)	105 (30.1)	625 (37.7)	0.87 (0.61-1.24)
Antipsychotics, *n* (%)	59 (16.9)	308 (18.6)	0.51 (0.25-1.05)
Mood stabilizers, *n* (%)	38 (10.9)	235 (14.2)	0.82 (0.51-1.31)
Psychotherapy, *n* (%)	108 (30.9)	451 (27.2)	0.72 (0.52-1.00)
Other therapy, *n* (%)^a^	54 (15.5)	137 (8.3)	1.50 (0.91-2.49)
Visits to psychiatrist, *n* (%)	291 (83.4)	1387 (83.6)	1.08 (0.74-1.58)
Visits to psychotherapist, *n* (%)	103 (29.5)	449 (27.0)	N/A
Visits to mental health worker, *n* (%)	62 (17.8)	181 (10.9)	N/A
Visits to other, *n* (%)	40 (11.5)	216 (13.0)	N/A

Abbreviations: *ADHD* = attention-deficit/hyperactivity disorder; *CI* = confidence interval; *DIVA* = Diagnostic Interview for ADHD in Adults; *N* = number of patients; *n* = number of affected patients; *N/A* = not available

^a^“Other therapy” refers to alternative therapeutic strategies, including osteopathy, yoga, psychoeducation, mindfulness training, etc

Note: Logistic regression was used for all binary outcomes

Overall, the percentage of patients receiving psychotherapy was similar between the ADHD and non-ADHD groups, but the odds ratio (OR) points toward lower use of psychotherapy for patients with ADHD (Table 4). Patients previously diagnosed with ADHD (but untreated) were less likely than patients without ADHD to have received psychotherapy in the previous 6 months (OR = 0.41 [95 % CI, 0.20-0.85]).

There was a trend toward greater use of “other” therapy by patients with ADHD (Table 4). Also, patients with previously diagnosed and treated ADHD were more likely to have received other therapy than patients with previously diagnosed but untreated ADHD (OR = 6.74 [95 % CI, 1.96-23.18]), patients whose ADHD was diagnosed during this study (OR = 5.11 [95 % CI, 2.58-10.13]), and patients without ADHD (OR = 4.88 [95 % CI, 3.03-7.86]).

Overall, ADHD and non-ADHD patients were similar in the number of visits to a psychiatrist (Table 4). However, patients with previously diagnosed and treated ADHD were more likely to have visited a psychiatrist in the previous 6 months than patients with previously diagnosed but untreated ADHD (OR = 3.51 [95 % CI, 1.38-8.92), patients whose ADHD was diagnosed during this study (OR = 2.97 [95 % CI, 1.38-6.39]), and patients without ADHD (OR = 2.36 [95 % CI, 1.19-4.70]).

### Patient disability and functioning

Patients with ADHD, as compared to patients without ADHD, had higher CGI-S scores (mean [95 % CI] 3.8 [3.7-3.9] versus 3.3 [3.2-3.3] points) and were more likely to be “moderately,” “markedly,” or “severely” ill (63.9 % versus 46.7 %). Among patients with ADHD, patients who were only diagnosed during the study were less ill (mean [95 % CI] CGI-S = 3.6 [3.4-3.8]) than patients previously treated for ADHD (mean [95 % CI] CGI-S = 4.1 [3.9-4.3]). On the SDS (Table 5), patients with ADHD, compared to patients without ADHD, reported more disability in all 3 areas (work/school, social life, and family life/home duties). Patients with ADHD also reported more days lost at work and more underproductive days. Among patients diagnosed with ADHD, differences in SDS mean (95 % CI) total scores between patients only diagnosed during this study (18.7 [17.8, 19.7]), those previously diagnosed and treated (19.1 [17.8, 20.3]), and those previously diagnosed but untreated (19.3 [17.4, 21.3]), were minimal. On the EQ-5D, patients with ADHD, as compared to patients without ADHD, reported more problems performing their usual activities and more often reported being “extremely anxious or depressed” (Table 5). More patients with newly diagnosed ADHD (due to the study) reported that they were “extremely anxious or depressed” (30.9 %) than patients with previously diagnosed with and treated for ADHD (15.8 %), and patients previously diagnosed with ADHD but untreated (21.3 %).

**Table 5:** SDS and EuroQoL-5 Dimensions Scores, by ADHD Diagnosis

	*N*	ADHD Diagnosis Established by the DIVA (DSM-5)	*N*	ADHD Diagnosis Not Established
		(*N* = 349)		(*N* = 1660)
SDS				
Total score, mean (SD)	348	18.9 (6.61)	1659	11.6 (8.55)
Work/school, mean (SD)	327	6.5 (2.62)	1466	4.1 (3.25)
Social life, mean (SD)	348	6.1 (2.68)	1659	3.8 (3.13)
Family life/home duties, mean (SD)	348	6.3 (2.61)	1659	3.8 (3.08)
Days lost, mean (SD)	348	1.9 (2.39)	1659	1.2 (2.14)
Underproductive days, mean (SD)	348	3.3 (2.46)	1659	2.0 (2.38)
EQ-5D				
Problems with mobility, *n* (%)	349	52 (14.9)	1660	285 (17.2)
Problems with self-care, *n* (%)	349	50 (14.3)	1660	141 (8.5)
Problems performing usual activities, *n* (%)	349	231 (66.2)	1660	684 (41.2)
Moderate/extreme pain or discomfort, *n* (%)	349	164 (47.0)	1660	790 (47.6)
Moderately/extremely anxious or depressed, *n* (%)	349	268 (76.8)	1660	1157 (69.7)
Visual analogue scale, mean (SD)	348	62.0 (22.86)	1659	64.3 (21.65)
Health state value, mean (SD)	348	0.609 (0.332)	1659	0.687 (0.292)

Abbreviations: *ADHD* = attention-deficit/hyperactivity disorder; *DIVA* = Diagnostic Interview for ADHD in Adults; *DSM* = *Diagnostic and Statistical Manual of Mental Disorders*; *EQ-5D* = EuroQoL-5 Dimension; *N* = number of patients; *n* = number of affected patients; *SD* = standard deviation; *SDS* = Sheehan Disability Scale

Patients with ADHD, compared to patients who did not suffer from ADHD, reported having fewer Master’s or Bachelor’s degrees and having more often obtained vocational training. Women with ADHD were less likely to be employed full time than women without ADHD; however, this effect of ADHD was absent for men. Patients with ADHD had more previous employers during the previous 3 years and were less likely to be working at their level of qualification. Patients with ADHD were also less likely to be living with a partner. In our sample, overall, patients with ADHD had fewer biological children. However, an age-adjusted analysis indicates that patients with ADHD had more biological children at 42 and 51 years of age. Patients with ADHD, compared to patients who did not have ADHD, were less likely to have a driving license at all, had their driving license revoked more often, and had more accidents, more speeding tickets, and more parking tickets (Table 6).

**Table 6:** Assessment of Functioning

	*N*	ADHD Diagnosis Established by the DIVA	*N*	ADHD Diagnosis Not Established	Odds Ratio or LS Mean Difference
		(*N* = 349)		(*N* = 1660)	(95 % CI)
Master’s/Bachelor’s degrees, *n* (%)		68 (19.4)		399 (24.0)	
ADHD, versus non-ADHD, OR (95 % CI)					0.60 (0.44-0.82)
Vocational training, *n* (%)		88 (25.2)		293 (17.7)	N/A
Total amount of formal instruction, years (SD)	346	12.58 (5.049)	1659	12.60 (4.586)	N/A
Student, n (%)		52 (14.9)		90 (5.4)	N/A
Employed full time, *n* (%)		106 (30.4)		580 (34.9)	
ADHD, versus non-ADHD (female), OR (95 % CI)					0.54 (0.36-0.81)
ADHD, versus non-ADHD (male), OR (95 % CI)					0.89 (0.62-1.26)
Number of different employers in last 3 years, mean (SD)	347	1.20 (1.598)	1659	0.90 (0.936)	N/A
Working at level of qualification, *n* (%)		159 (45.6)		840 (50.6)	N/A
Number of biological children, mean (SD)	348	0.93 (1.230)	1655	1.13 (1.135)	
ADHD versus non-ADHD (at age 31), LS Mean difference					0.10 (−0.03-0.23)
ADHD, yes versus non-ADHD (at age 42), LS Mean difference					0.20 (0.07-0.33)
ADHD, yes versus non-ADHD (at age 51), LS Mean difference					0.28 (0.10-0.47)
Age when first biological child was born (years), mean (SD)	159	25.50 (5.685)	998	26.70 (5.574)	N/A
Age when first biological child was born (<18 years), *n* (%)		7 (2.0)		23 (1.4)	N/A
Living with parents, *n* (%)		78 (22.3)		215 (13.0)	
ADHD versus non-ADHD, OR (95 % CI)					1.09 (0.76-1.57)
Driving license held, *n* (%)		239 (68.5)		1303 (78.5)	N/A
Number of times license revoked in last 3 years, mean (SD)	239	0.07 (0.303)	1300	0.04 (0.222)	N/A
Number of speeding tickets in last 3 years, mean (SD)	239	1.05 (2.072)	1299	0.55 (1.664)	N/A
Number of parking tickets in last 3 years, mean (SD)	239	1.12 (3.299)	1296	0.60 (1.818)	N/A
Number of car accidents in last 3 years, mean (SD)	239	0.31 (0.808)	1301	0.16 (0.555)	N/A
Frequency of debt, *n* (%)		124 (35.5)		404 (24.3)	N/A
Prosecuted since adulthood, *n* (%)		48 (13.8)		102 (6.1)	N/A
Current tobacco use, *n* (%)		165 (47.3)		612 (36.9)	N/A

Abbreviations: *ADHD* = attention-deficit/hyperactivity disorder; *DIVA* = Diagnostic Interview for ADHD in Adults; *N* = number of patients; *n* = number of affected patients; *SD* = standard deviation

Note: Logistic regression was used for all binary outcomes; number of biological children was analyzed using analysis of covariance

## Discussion

To our knowledge, this is the first multinational, cross-sectional study to evaluate the prevalence of ADHD in outpatient general psychiatric care in Europe. Given the high rate of psychiatric comorbidities in adult individuals with ADHD [1, 2], it seems logical that many individuals with ADHD would already have had contact with psychiatric services and would therefore be likely to be receiving psychiatric care in outpatient clinics. We found the prevalence of ADHD in our sample, based on DSM-IV-TR criteria, to be 15.8 %. However, based on the new DSM-5 criteria, as expected, the prevalence rate in our sample increased to 17.4 %, overall. As we hypothesized, this value is much higher than for the general population, for which there are several estimates, including 2.5 % [14], 3.4 % [13], and 4.4 % [15]. Our estimate agrees well with Almeida Montes and colleagues [16]^16^ who found a prevalence of 16.8 % in nonpsychotic outpatients. However, they found the rate of ADHD was more than twice as high for women than for men (21.6 % versus 8.5 %), the reverse of what we found. (It should be noted, however, that a higher proportion of women were newly diagnosed with ADHD in the present study.) Rao and Place [17] reported that 22 % of their sample from outpatient clinics in North East England could be diagnosed with ADHD, which is higher than our overall prevalence estimate but in good agreement with our estimate for psychiatric outpatients in the United Kingdom (22.4 %).

We found that the most common subtype of ADHD in our sample was the combined subtype (63.2 %). However, a recent meta-analytic review found that the most common form of ADHD in the general adult population is the inattentive type [27]. It is possible that the combined subtype only appears to be more prevalent because treatment is more often sought for these patients. Alternatively, the combined subtype may carry a greater risk for psychiatric comorbidity [28].

Overall, we noted considerable variability in prevalence of ADHD associated with country, possibly reflecting differences in the referral selection process in their respective health care systems.

But what is even more relevant to patients, their treating mental health professionals, and the respective national health care system, is the impact of ADHD, in terms of well-being, functioning, and impairment. We found that, compared with patients who did not have ADHD, patients with ADHD had higher CGI-S scores (were more ill), which is an indicator of functional disability, as well. This observation was affirmed in that they reported more problems performing their usual activities on the EQ-5D, and more disability overall. The SDS results indicate that, on average, patients with ADHD have moderate to severe impairments in work, family, and social functioning, while the impairment of the other outpatients without ADHD is between mild and moderate. The high level of impairment may be related to the fact that ADHD is causing a chronic impairment in multiple domains, in contrast to episodic impairment in depression and impairment limited to one or a few domains as is usually the case with anxiety disorders [18]. In addition, the fact that ADHD starts in childhood and can lead to multiple occurrences of failure and social distress could give reason to the observed developments.

In agreement with previous studies from various geographic areas [6, 13, 29–31], we found that outpatients with ADHD were more likely to report substance abuse/dependence than patients who did not have ADHD. A recent meta-analysis found that the prevalence of ADHD in patients with substance use disorder was much higher (23.1 %) than in the general population [30]. It has also been reported that people with ADHD are more likely to have a substance use disorder than control patients without neuropsychiatric conditions [29, 31]. Importantly, there is evidence that the risk of substance use disorder is reduced as a result of ADHD treatment [32]. Previous studies have also shown that ADHD is often associated with a number of comorbid psychiatric disorders, such as mood and anxiety disorders or substance use disorders [13]. We found that patients who had ADHD that had been diagnosed and treated before study entry suffered from anxiety to a lesser degree (28.1 %) than those diagnosed with ADHD but untreated (44.7 %). This could be another indicator that timely treatment of ADHD could reduce the burden of psychiatric disease. In our sample, 88.5 % of outpatients with ADHD had at least one additional psychiatric diagnosis.

We also found that patients with ADHD tended to have lower academic achievement and were less likely to be employed full time (women only), even in comparison to patients with other psychiatric disorders. Though these results may be partially explained by the younger age of the patients with ADHD, we also found that those patients were more likely to have received vocational training than patients who did not have ADHD. These findings support earlier work showing that adults with ADHD, compared with controls, trended toward having lower occupational attainment and had significantly more academic problems in school [33].

These data are also in line with earlier reports that adult patients with ADHD tend to have lower socioeconomic status [1], more functional impairment [2, 34], and a diminished quality of life [3–5], even more than patients in psychiatric outpatient care. This is a compelling argument to screen patients presenting at psychiatric outpatient facilities for ADHD and to offer individuals with confirmed ADHD who show impairments the appropriate care for their ADHD symptoms in addition to those of their comorbid diagnoses.

Finally, we found that the prevalence of ADHD was slightly higher (17.4 % versus 15.8 %) according to DSM-5 criteria, as compared to DSM-IV-TR criteria. A similar conclusion was made in a study of patients seeking treatment for substance use [35]. However, this higher prevalence was endorsed by the clinical opinion of the participating investigators, suggesting that the new DSM-5 criteria are matching better the clinical assessment than the DSM-IV-TR criteria.

The prevalence of ADHD in the general psychiatric outpatient population is so high that systematic screening for it is recommended, especially since the symptoms and complaints may relate to comorbid disorders so severe that genuine ADHD symptoms are masked. However, in this population the specificity of the common screening tools, like the ASRS, are weak, with only half of the patients screening positive having a confirmed diagnosis. Therefore, a thorough diagnostic assessment of ADHD, like we did with the DIVA instrument, is necessary. Because such an assessment takes a considerable amount of time (at least 1 hour per patient), it would be very useful to look for additional screening criteria that could improve the specificity of the existing tools with minimal loss of sensitivity.

### Limitations

First, as this was an observational study, we cannot draw any conclusions regarding causality. We can present data regarding how certain patient characteristics are associated with an ADHD diagnosis, but we do not know if ADHD caused or contributed to any of those characteristics.

Secondly, we have a substantial sample size for Europe overall, but our ability to make comparisons between countries is limited because of the small sample size for some countries and because settings, which had their own variability, were different between countries. However, our study procedures were set up to minimize selection effects by approaching each and every patient attending a study facility during carefully specified times. Careful attention was given to site selection for each country with the intention that the patients were representative. However, for some countries, because of the limited number of sites able to participate, the results may be less representative. In addition, variability in training/experience in assessing patients may have affected estimates of ADHD prevalence in different centers and countries.

Third, other studies have shown that patients with ADHD tend to underreport lifetime inattention problems when giving their history and when completing scales [36]; however, we attempted to minimize this effect by including reports from family members. Another limitation concerns the 265 patients who screened positive for ADHD, but for whom the DIVA questionnaire was not performed for various reasons. However, a sensitivity analysis showed that this had a relatively minor impact on the overall study.

Fourth, the high exclusion rate brings into question the generalizability of our estimates of ADHD prevalence. However, there appeared to be no relationship between exclusion rates and ADHD prevalence when measured by country.

Fifth, patients who screened negative were not further tested using the DIVA, so we do not have an estimate of false negatives. However, the ASRS is a very sensitive instrument, so false negatives should have been minimal. In addition, a positive screen on the PDI-4 ADHD questions, a previous ADHD diagnosis, or suspicion of ADHD by clinical impression also resulted in a positive screen, further reducing the chances of false negatives.

## Conclusions

Our findings indicate that ADHD is present in a substantial proportion of nonpsychotic patients seeking psychiatric help. Compared to other psychiatric outpatients, they are among the most impaired and their medical resource needs are comparable and in some aspects higher than in patients without ADHD. It is hoped that data from this study contribute to a better understanding of the presence, the pattern of clinical presentation, and clinical picture of patients and of implications of ADHD in adult mental healthcare. This knowledge may be the basis for a better allocation of appropriate diagnostic and treatment resources and lead to a reduction of primary and secondary costs due to a more focused treatment once the diagnosis of ADHD can be properly confirmed or dismissed. Proper application of these findings may also result in a reduced burden to the patient due to earlier therapeutic interventions and as a consequence possibly better outcomes for patients with ADHD.

### Ethics committees

Ethics commission, Medical University Vienna, Borschkegasse 8b/6, A-1090 Vienna, Austria

Ethics commission for the state, Salzburg, SEBASTIAN-STIEF-GASSE 2, 5010 SALZBURG, Austria

Ethics commission of the city Vienna, Thomas-Klestil-Plaz 8, A-1030 Vienna, Austria

Ethics commission for the state, Niederösterreich, Landhausplatz 1, Haus 15B, A-3109 St. Pölten, Austria

AZ Sint-Lucas Brugge, Ethisch Comité, Sint-Lucaslaan 29, 8310 Brugge, Belgium

Sint-Andriesziekenhuis Tielt, Lokale Commissie voor Ethiek, Krommewalstraat 11, 8700 Tielt, Belgium

Algemeen Stedelijk Ziekenhuis - Campus Aalst, Ethisch Comité/ Dr. Fosselle, Merestraat 80, 9300 Aalst, Belgium

Freiburger Ethik-Kommission GmbH International, Mozartstraße 21, 79104 Freiburg, Germany

Ethikkommission der Fakultät für Medizin der Technischen Universität München, Ismaninger Straße 22, 81675 Munich, Germany

Ethikkommission an der Medizinischen, Fakultät der Universität Rostock, St.-Georg-Str. 108, 18055 Rostock, Germany

Otto-von-Guericke-Universität Magdeburg, Medizinische Fakultät, Universitätsklinikum Magdeburg A. ö. R., Ethikkommission, Leipziger Straße 44, 39120 Magdeburg, Germany

IRBN, Postbus 85, 6600 AB Wijchen, Netherlands

EC in Stockholm, Sweden

CEIC Hospital U. Príncipe de Asturias, Secretaría Técnica, Ctra. Alcalá-Meco s/n 28805 Alcalá de Henares, Madrid, Spain

CEIC Unidad de Investigación e Innovación/CEIC FHAG, Fundación Hospital Asil de Granollers, Av. Francesc Ribas s/n, 08402 Granollers, Barcelona, Spain

CEIC Hospital Virgen de la Salud., Comité Ético de Investigación Clínica, Antigua Escuela de Enfermeras, Mª Ángeles Jimenez, C/ Alicante, s/n,1ªplanta-Despacho 4, 45005 Toledo, Spain

CEIC de Euskadi, Dirección de Farmacia, Departamento de Sanidad, C/Donostia-San Sebastian, n°1, 01010 Vitoria- Gobierno Vasco, Spain

CEIC Consorci Sanitari de Tarrasa, CONSULTA MEDICA CERCLE EGARENC., C/San Pere, 48. 3ªplanta, 08221-Tarrasa, Spain

CEIC Central de Asturias, Centro Saludo Mental Siero., C/Maestro Martín Galache, SN., Pola de Siero. Asturias, Spain

CEIC Hospital Ramón y Cajal. Planta −2 Dcha. Carretera Colmenar km 9.100, 28034 MADRID, Spain

NHS, Health Research Authority, NRES Committee South Central - Berkshire B, Bristol REC Centre, Whitefriars, Level 3, Block B, Lewins Mead, BS1 2NT Bristol, United Kingdom

## Abbreviations

ADHD: Attention-deficit/hyperactivity disorder
ASRS: Adult Attention-Deficit/Hyperactivity Disorder Self-Report Scale
CGI-S: Clinical Global Impressions-Severity
DIVA: Diagnostic Interview for Attention-Deficit/Hyperactivity Disorder in Adults, version 2.0
DSM-5: Diagnostic and Statistical Manual of Mental Disorders, 5th Edition
DSM-IV-TR: Diagnostic and Statistical Manual of Mental Disorders, 4th Edition, Text Revision
ERB: Ethical review board
EQ-5D: EuroQol-5 Dimensions
EQ VAS: EuroQol Visual Analog Scale
GCP: Good Clinical Practice
OR: Odds ratio
PDI-4: Provisional Diagnostic Instrument-4
QoL: Quality of life
SDS: Sheehan Disability Scale
